# Long-Term Dynamical Constraints on Pharmacologically Evoked Potentiation Imply Activity Conservation within *In Vitro* Hippocampal Networks

**DOI:** 10.1371/journal.pone.0129324

**Published:** 2015-06-12

**Authors:** Mark Niedringhaus, Xin Chen, Rhonda Dzakpasu

**Affiliations:** 1 Interdisciplinary Program in Neuroscience, Georgetown University Medical Center, Washington, District of Columbia, United States of America; 2 Department of Physics, Georgetown University, Washington, District of Columbia, United States of America; 3 Department of Pharmacology and Physiology, Georgetown University Medical Center, Washington, District of Columbia, United States of America; SUNY Downstate MC, UNITED STATES

## Abstract

This paper describes a long-term study of network dynamics from *in vitro*, cultured hippocampal neurons after a pharmacological induction of synaptic potentiation. We plate a suspension of hippocampal neurons on an array of extracellular electrodes and record electrical activity in the absence of the drugs several days after treatment. While previous studies have reported on potentiation lasting up to a few hours after treatment, to the best of our knowledge, this is the first report to characterize the network effects of a potentiating mechanism several days after treatment. Using this reduced, two-dimensional *in vitro* network of hippocampal neurons, we show that the effects of potentiation are persistent over time but are modulated under a conservation of spike principle. We suggest that this conservation principle might be mediated by the appearance of a resonant inter-spike interval that prevents the network from advancing towards a state of hyperexcitability.

## Introduction

One of the major challenges for the brain is to maintain a stable operating state while retaining sufficient flexibility to grow [1, 2]. However, neural systems must experience plasticity in response to external stimuli and adapt to its environment. How these two opposing constraints reconcile is not well understood. During development, a neural circuit will undergo enormous changes in activity. There is a large degree of spontaneous activity as the immature circuit is largely excitatory, with strong recurrent connectivity [3–6]. Subsequently there is a shift and the GABAergic neurons transition from excitatory to inhibitory [6, 7]. The adult brain must also be receptive to experience-dependent plasticity. The brain still receives sensory inputs for which it may be required to adapt to a new stimulus. The operating state of the brain must be such that it does not produce excessive excitation or insufficient excitation so that the brain is able to perform its normal functions as well as preserve changes that occur from experiential learning.

Modulations in excitability can occur via an increase in synaptic efficacy and a common method is synaptic potentiation. One example is long-term potentiation (LTP), which can be described by the synaptic strengthening that is induced by the coordinated spiking of pre- and postsynaptic neurons, thereby increasing the likelihood of the production of an action potential [8–15]. This synaptic potentiation is thought to form the cellular mechanism for learning and memory and is frequently induced via electrical stimulation [16–19]. However electrical stimulation is spatially restricted which will therefore limit the number of synapses for which plasticity changes can be induced. Studying the effects of synaptic potentiation in a large population of synapses permits the assessment of a network response to a small spatial scale perturbation as well as allowing for extensive biochemical investigations of LTP. As a result, chemical methods have been introduced to induce synaptic potentiation in a large population of synapses and it has been demonstrated that several of these methods are evoked via similar biochemical pathways that have been elucidated in the electrical techniques [20,21,22].

However, while increasing synaptic efficacy leads to an increase in activity, what can happen in the long term? If left unchecked, a network might evolve into a pathological state of uncontrolled excitation, at one extreme, that could result in epileptiform activity or the other extreme, a cessation of activity. Therefore, the network, while still experiencing activity-dependent plasticity, needs to operate within a well-regulated regime to prevent an untenable outcome. Regulatory mechanisms are needed to balance the effects of increased excitability.

We previously reported on changes in network activity from short-term studies in which we used a pharmacological paradigm to potentiate synapses within an *in vitro* network of hippocampal neurons [23, 24]. After twenty minutes of treatment we saw a dramatic and persistent network-wide increase in firing rates. We also showed that the concomitant bursting frequency increased, with more spikes that are not within bursts recruited into the burst profile. This evoked an elevation of network activity that is reminiscent of attractor dynamics. In our current studies we use the same paradigm to potentiate synapses within an *in vitro* network of hippocampal neurons. As before, we use this paradigm to study how increasing excitability can modulate activity but here we focus on the long-term impact of potentiation on dynamical activity.

We use a multi-electrode array (MEA) to record changes in extracellular potentials, specifically action potentials from capacitively-coupled neural units. MEAs facilitate the characterization of spiking activity from *in vitro* networks of neurons and also allows for long-term studies [25–31]. We treat cultured networks of hippocampal neurons with a cocktail of two drugs in order to induce synaptic potentiation: forskolin, which activates adenylyl cyclase, and rolipram, which is a phosphodiesterase inhibitor [21]. Together, this cocktail increases the levels of cyclic AMP thereby potentiating a large fraction of synapses in the network. This results in an increase in the probability of neuronal spike generation. We show that there is a considerable reorganization of spiking activity one day after treatment that consists of long periods of high frequency firing. Periods of quiescence follow and the activity during quiescence decreases over time. Additionally we observe that the networks do not evolve towards a state of hyperexcitability as there appears to be a mechanism in which some channels maintain elevated spiking activity and others decrease activity. After the initial increase in activity, overall spiking returns to levels observed in the unperturbed, control networks. Lastly we show the rise of a peak in the distribution of inter-spike intervals around 100 ms and we suggest that this might be a resonant interval that maintains spike conservation.

## Methods

### 1. Cell culture

#### Ethics Statement

All experimental procedures were approved by the Georgetown University Animal Care and Use Committee (GUACUC). We used a protocol modified from [32] to extract hippocampal tissue from embryonic day 18 (E18) Sprague-Dawley rats. Briefly, the neural tissue was finely chopped and digested with 0.1% trypsin followed by mechanical trituration. Upon reaching a single cell suspension, approximately 200,000 cells were added to each multi-electrode array (MEA, Multi Channel Systems MCS GmbH, Reutlingen, Germany) that was previously treated with poly-d-lysine and laminin (Sigma, St. Louis, MO). This results in an approximate plating density of 600 cells/mm^2^. Cultures were maintained in Neuralbasal medium with B27 (Invitrogen, Carlsbad, CA) and kept in a humidified 5% CO_2_ and 95% O_2_ incubator at 37°C. To provide a consistent supply of nutrients, one third of the media was changed on a bi-weekly basis.

### 2. Electrophysiological Recordings

We recorded all spontaneous electrical activity using a multi-electrode array (MEA). The MEA is composed of 59 titanium nitride electrodes, one reference electrode and four auxiliary analog channels each of which is 30 μm in diameter. These electrodes are arranged on an 8x8 square array with an inter-electrode spacing of 200 μm. Within a few hours after plating, the cells in suspension settle and adhere to the silicon nitride substrate of the MEA. After seven days spontaneous electrical activity is detectable. We use the MEA1060 preamplifier and sample electrical activity at a 10kHz acquisition rate to allow for the detection of multi-unit spikes. The data is digitized and stored on a Dell personal computer (Round Rock, TX). Possible exposure to contaminants and fluctuations in osmolality and pH were significantly reduced during the data acquisition period by the use of an MEA cover made of a hydrophobic membrane [33]. This membrane provides a seal that is semi-permeable to CO_2_ and O_2_ and is largely impermeable to water vapor. Experiments from at least four MEAs for each condition, including vehicle and control cultures, were performed on a heated stage at 37°C at 14 days *in vitro* (14DIV), a time point during development in which the network displayed vigorous spontaneous electrical activity and for which network connectivity is well-established [28]. To ensure reproducibility of results across animals, all reported experimental groups were comprised of multiple cultures derived from multiple experimental preparations. Results obtained from cultures within and across different preparations were not significantly different.

### 3. Synaptic potentiation induction

We induced synaptic potentiation within cultured networks of hippocampal neurons using forskolin (50 μm) and rolipram (100 nM) on DIV14 as this paradigm has been previously shown to induce chemical LTP (21). Forskolin was dissolved in dimethyl sulfoxide (DMSO) to a stock concentration of 50 mM. Rolipram was dissolved in DMSO to a stock concentration of 100 μM. All reagents were purchased from Sigma-Aldrich (St. Louis, MO). After a pre-treatment recording of 20 minutes of spontaneous activity, the MEA was removed from the recording stage. The conditioned media was aspirated from the MEA and stored in the incubator until after the treatment. The cells were then washed three times with 200 μL of artificial cerebrospinal fluid (ACSF; 140mM NaCl, 5 mM KCl, 1.5 mM CaCl_2_, 0.75 mM MgCl_2_, 1.25 mM NaH_2_PO_4_, 20 mM glucose, 15 mM HEPES/NaOH adjusted to 7.4 pH) and then treated with 400 μL of ACSF containing 50 μM forskolin and 0.1 μL rolipram. After 15 minutes, the treatment solution was removed and the cells were washed three times with 200 μL of unconditioned media. The original conditioned media was then added back to the cell culture in the MEA and the MEA was returned to the incubator. Long-term effects of synaptic potentiation were assessed 24 and 96 hours after treatment.

This experimental design includes two control groups: a “vehicle” group in which 1 μL of DMSO was diluted into the conditioned media to control for effects of solvent and mechanical perturbation introduced by solution exchange, and a “control” group to account for the developmental changes of the unperturbed culture. For these groups, no treatment or washing was performed, and recordings were performed at the same time points as the other two groups.

### 4. Data Analysis

To obtain a stable baseline for the spike threshold procedure, we removed low frequency fluctuations by high-pass filtering all voltage traces at 200 Hz. Extracellularly recorded spikes were detected using a threshold algorithm from Offline Sorter (Plexon Inc., Dallas TX). The threshold is calculated as a multiple of the standard deviation, 5σ, of the biological noise. No attempt was made to discriminate and sort spikes within each electrode because for this study, we concentrate on overall network spiking activity and the signal from each electrode suitably reflects these dynamics. This spike identification process results in an M x N matrix where M corresponds to the electrode number and the N’s are the time stamps of the spikes.

We used custom-written software written in MATLAB (The MathWorks, Natick, MA) to analyze the network activity recorded from the cultured hippocampal networks. We plotted the number of spikes from each electrode in the pre-treatment recordings against the spike counts one and four days after synaptic potentiation. The time evolution of these spike distributions was compared to those within the vehicle and control networks. We also generated log histograms of the inter-spike intervals (ISIs) from each MEA. This was this done by computing ISIs for each individual electrode within a given MEA and pooling them into a single distribution to distinguish between populations of ISIs that participate within brief episodes of spiking activity (short time-scale ISIs) from those ISIs that represent intervals between periods of high activity (long time-scale ISIs). The log histograms were used to characterize aspects of the high frequency activity observed within the potentiated networks.

Next, we investigated the variability of firing rates, FR, within each network. We calculated the Fano Factor, defined as [34]:

$$\text{FF}=\frac{\text{var}<\text{FR}>}{<\text{FR}>}$$

When the Fano factor is equal to unity, the electrodes exhibit dynamics of Poisson processes. A Fano factor between 0 and 1 is under-dispersed and suggests that the distribution has a more regular firing pattern than that of a Poisson distribution. If the Fano factor is greater than 1, the distribution is over-dispersed and indicates large fluctuations of spiking activity throughout the epoch of activity.

Lastly, we investigated the time evolution of oscillatory activity within the networks. We used a short time, Fourier transform based time-frequency analysis (STFT—The Mathworks, Natick, MA). For this analysis, we returned to the unfiltered data sets in order to study both low- and high-frequency oscillations. For the low-frequency oscillations, we performed a low-pass filter with a cutoff at 250 Hz. For the high-frequency oscillations, we performed a high-pass filter with a 200 Hz floor. The STFT analysis will result in a power spectrum as a function of time in which the power at a particular frequency will be displayed as a color map.

## Results

**Fig 1:**
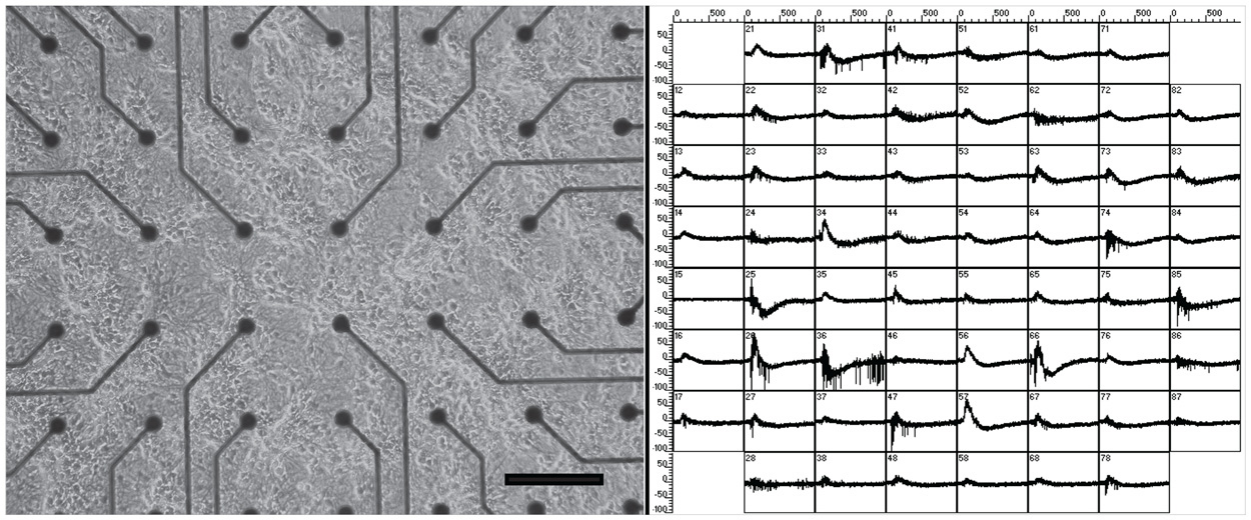
Hippocampal neurons cultured on multi-electrode arrays. Left: A DIC image of cells plated on the MEA. Scale bar = 200 μm Right: Screen shot of raw, unfiltered data of spontaneous activity from the networks. Each box represents one second of activity.

Fig 1, left, shows a differential interference contrast (DIC) micrograph of a 14DIV culture of hippocampal neurons plated on the multi-electrode array. On the right is an accompanying screen shot of network electrical activity from the culture. Each box corresponds to one-second of activity. The dynamics consist of a rich mix of high- and low-frequency spikes.

### 1. Raster Plots of Evolving Network Activity

**Fig 2:**
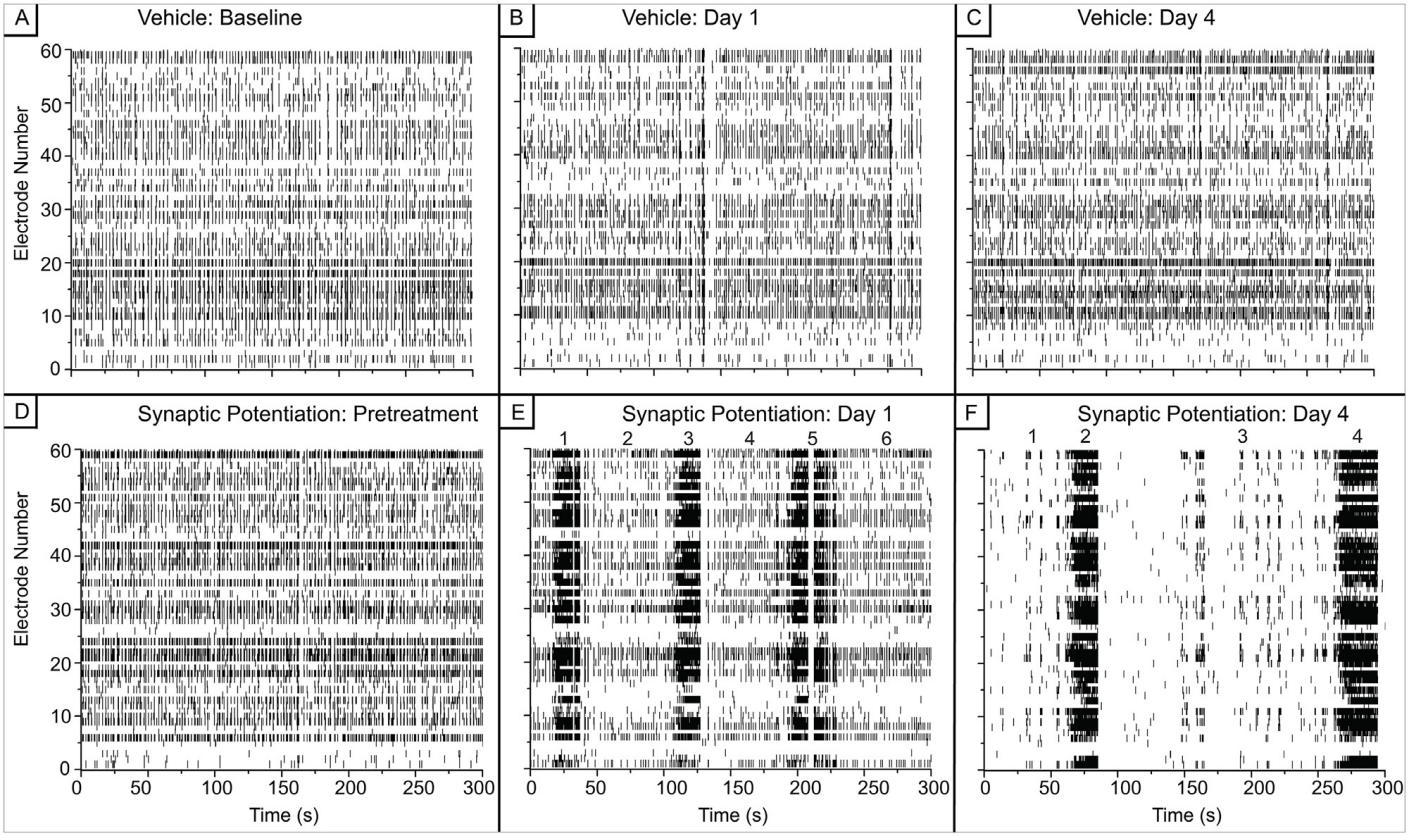
There is a reorganization of network spiking activity after synaptic potentiation. A-C) Representative raster plot of network activity from a vehicle MEA is largely uniform over the three days. D-F) Representative raster plot of network activity from an MEA after treatment shows long epochs of high frequency spiking activity one and four days after treatment. These epochs are separated by periods of quiescence during which the activity decreases over time.

Fig 2 displays representative raster plots of five minutes of activity from the treated and vehicle networks. Dynamics from the vehicle experiments appear to be quite uniform over the course of the five-day period (Fig 2A–2C). In contrast, the activity from the synaptic potentiation-treated networks changes considerably (Fig 2D–2F). Initially, the pattern observed within the pre-treated activity is very similar to the pre-treatment activity in the vehicle networks. However, one day after the induction of synaptic potentiation, the spiking activity organizes into long epochs, i.e., super-bursts, of intense activity. This becomes more pronounced four days after treatment. The spiking activity in the intervals between these intensely firing epochs re-structures—the period prior to these long epochs begins to cluster and organize (Fig 2F) and there is less activity between these long epochs that in the vehicle or pre-treatment conditions.

**Fig 3:**
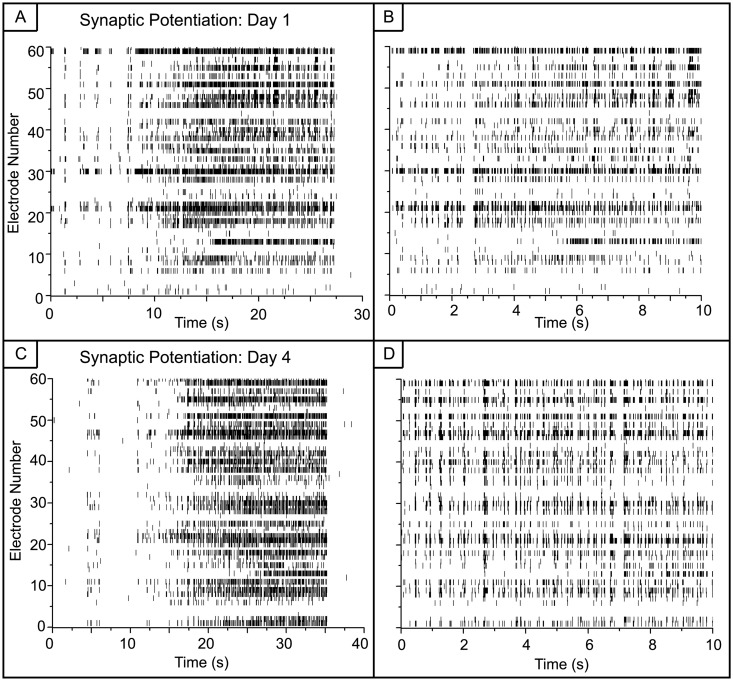
Expanded view of activity from synaptically potentiated networks. A) C) A 30-second window extracted from the epoch of high activity seen in Fig 2E and 2F. At the onset of these long epochs, there are several short bursts of activity. B) D) A 10-second window extracted from the epoch of high activity seen in A and C. During these long epochs, the activity is mostly tonic.

**Fig 4:**
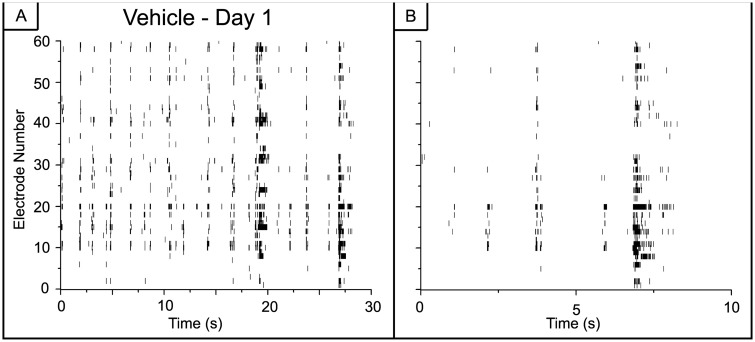
Vehicle networks do not show large changes in spiking activity. A) Activity is clustered into short bursts with no transition to high frequency tonic activity at the shorter time scale displayed in panel B. Spiking activity on day 4 is very similar to activity on day 1 (data not shown).

When the activity is examined in finer temporal detail as shown in Fig 3, the dynamics appear to be a mix of tonic firing of individual spikes and small bursts. Preceding the large, super-bursts of activity are short episodes of bursts with diminishing inter-episode intervals (Fig 3A and 3C). Within the large super-bursts (Fig 3B and 3D), a primarily tonic activity pattern is present. In contrast, there appears to be clusters of short bursts in the vehicle networks with no transition to high frequency tonic activity (Fig 4A and 4B).

**Fig 5:**
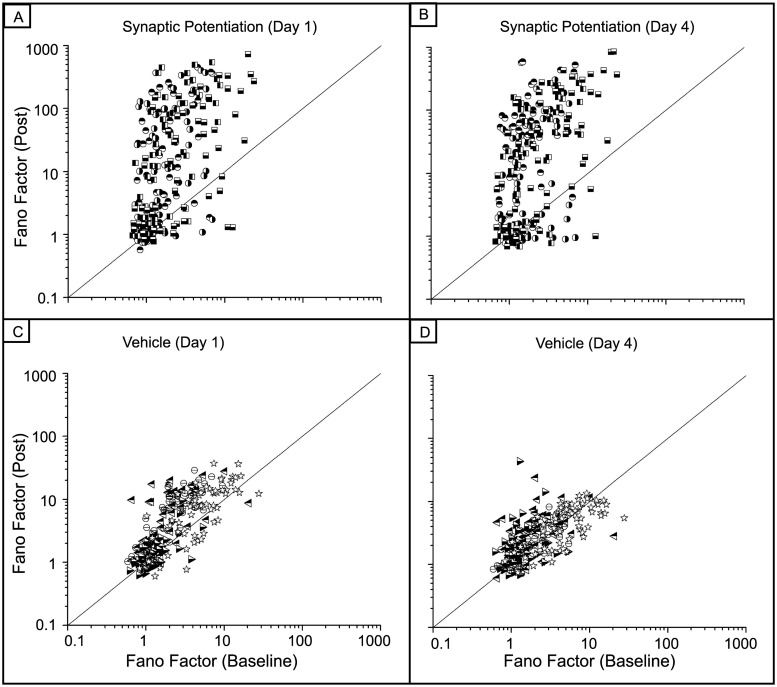
There is a large increase in the Fano Factor after synaptic potentiation. A) B) Spiking activity within networks that experienced synaptic potentiation become more variable after potentiation. This is due to the different periods of high frequency spiking punctuated by periods of quiescence. One-way ANOVA, p < 10^–8^. C) D) In contrast, the Fano factor for the vehicle networks remains largely unchanged as the networks mature.

**Fig 6:**
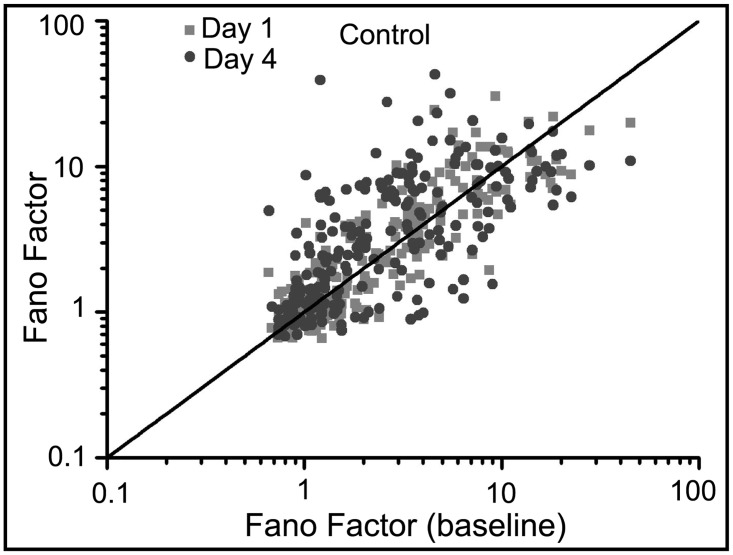
Fano factor does not change in the control networks. The values of the Fano factor are primarily distributed along the identity line and appear to be unchanged as the networks mature. (one-way ANOVA, p < 10^–6^)

To investigate the regularity in the spiking pattern, we calculated the Fano factor (Figs 5 and 6). There is a large increase in the Fano factor one and four days after the synaptic potentiation-treatment (one-way ANOVA, p < 10^–8^). This increase reflects the large variablilty in the evolving spiking dynamics. The activity alternates between long epochs of bursts of bursts and periods of shorter spiking episodes interspersed with quiescence as visualized in the raster plots of Figs 2 and 3. In contrast, values of the Fano factor for both the vehicle and control networks fall largely along the identity line suggesting that the variability in firing patterns is low and does not change as a function of time. Additionally, mechanical perturbations as well as network maturation do not appear affect the pattern of activity within these networks (one-way ANOVA, p < 10^–6^).

### 2. Network Spiking Activity

**Fig 7:**
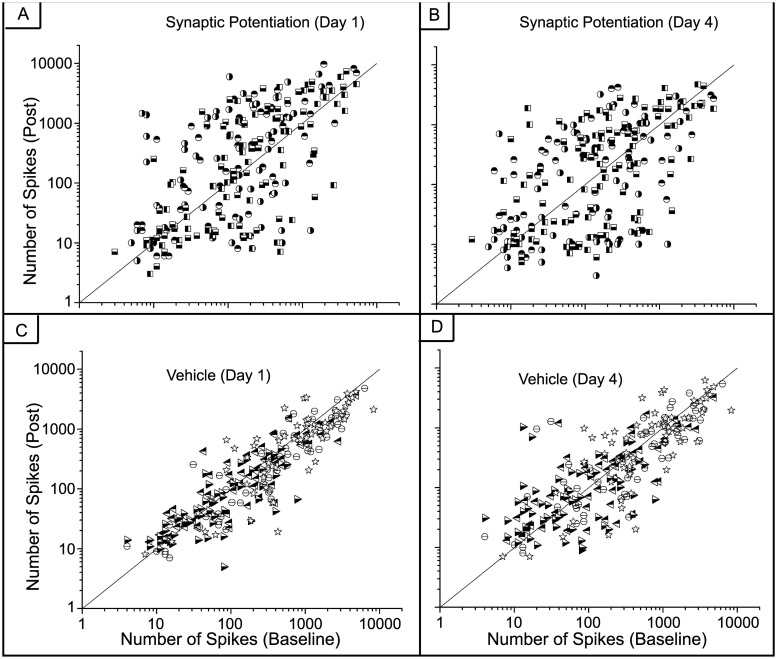
Not all electrodes maintain an increase in spiking activity after synaptic potentiation. Number of spikes before and after treatment. Each symbol corresponds to an electrode from a different MEA. A) Synaptic potentiation experiments. Each symbol represents an electrode from each MEA. (N = 4) A uniform expansion is observed and takes the shape of an ellipsoid with principle axis of rotational symmetry about y = x. (one-way ANOVA, p <10^–5^) B) Vehicle experiments. (N = 4) The change in the shape is negligible. (one-way ANOVA, p < 10^–6^)

**Fig 8:**
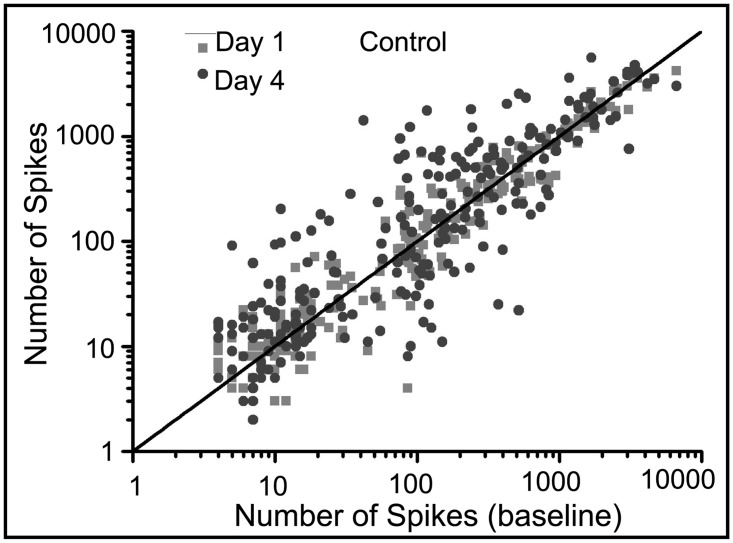
Number of spikes in control hippocampal networks show little change. (one-way ANOVA, p< 10^–6^) Fluctuations about the identity line are a result of network maturation.

**Fig 9:**
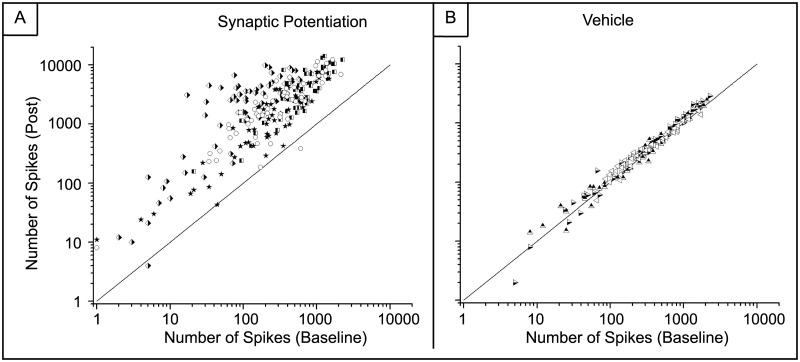
There is a persistent increase in spiking activity after synaptic potentiation. A) Spike counts from all electrodes before and treatment. Most electrodes have an increase in activity with a large cluster displaying an increase of at least two orders of magnitude. (one-way ANOVA, p<10^–9^) B) Spike counts from the DMSO-treated MEAs show no increase in activity. (one-way ANOVA, p<10^–7^). Each symbol corresponds an electrode from a different MEA. Three MEAs were used for the vehicle and four MEAs were used for the synaptic potentiation studies. The diagonal line denotes the identity line, y = x. (Modified and reprinted with permission.)

Fig 7 shows the number of spikes from each electrode within each MEA before treatment (baseline) plotted on a log scale against the number of spikes from each electrode one and four days after treatment as well as within the vehicle networks. Activity increases observed after synaptic potentiation were maintained for a large number of electrodes but there is also a considerable number of electrodes for which electrical activity decreases (Fig 7A and 7B) (one-way ANOVA, p <10^–5^). This is evident by the symmetric expansion about the identity line (y = x). This expansion is much reduced within the vehicle (Fig 7C and 7D) and control (Fig 8) networks. In contrast, Fig 9 shows our previously published results displaying the number of spikes from each electrode 20 minutes after induction of synaptic potentiation. In those experiments, most of the electrodes show a marked increase in activity (one-way ANOVA, p <10^–6^).

**Fig 10:**
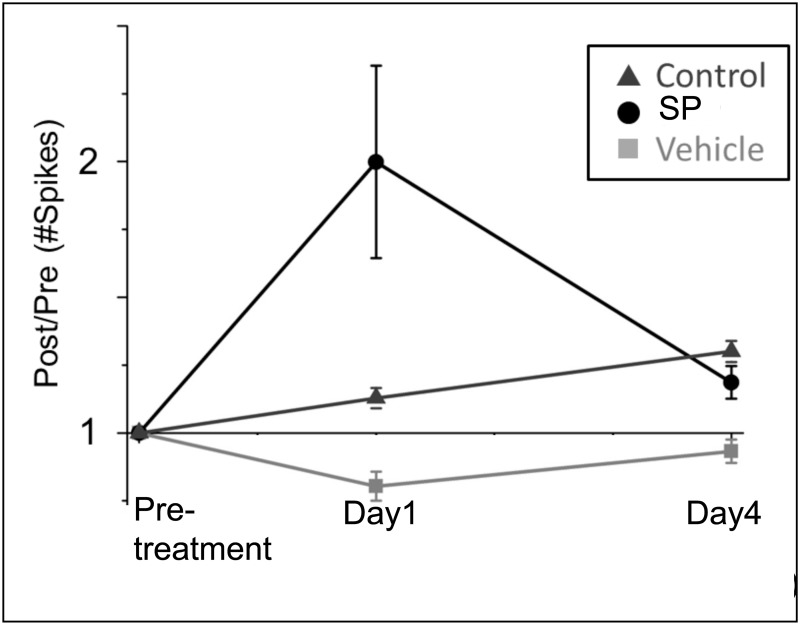
There is an increase in network spiking activity one day after synaptic potentiation but activity returns to the control curve three days later (one-way ANOVA, p<10^–9^). In contrast, there is a graduate increase in activity for the control networks. There is a small decrease in activity within the vehicle networks, which is likely due to mechanical perturbations (one-way ANOVA, p <10^–7^). However, activity increases thereafter with a similar slope to that of the control curve.

The impact of synaptic potentiation is further demonstrated by the sharp increase in the fold-change in activity one day after the synaptic potentiation application (Fig 10). While there is a marked increase in activity one day after treatment, overall activity returns to the average number of spikes generated in the control networks (one-way ANOVA, p <10^–9^). The increase in the control networks represents network maturation due to the increase in connectivity during development [28, 35]. In comparison, there is a slight decrease in activity within the vehicle networks on day 1 and those dynamics return to baseline activity by day 4 (one-way ANOVA, p<10^–7^). This offset of spiking activity is likely due to mechanical perturbations introduced by solution exchange but we note that the slope for the vehicle networks after day 1 is very similar to that of the control networks. Lastly, these mechanical influences do not appear to alter the distribution of spike counts as the network develops; activity from the vehicle networks on both day 1 and day 4 with respect to pre-treatment activity largely follows the identity line (Fig 7C and 7D) and again are very similar to the control networks in Fig 8.

**Fig 11:**
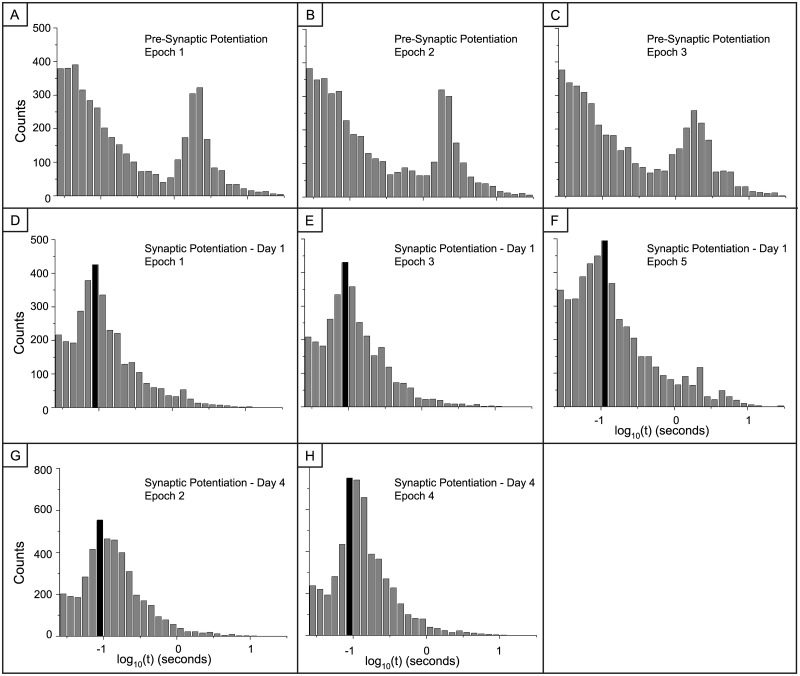
Log(ISI) histograms of activity within epochs of activity before and after synaptic potentiation. A-C: Activity from the pre-treatment MEAs in Fig 2D was equally divided and the ISI histograms are presented. There are no changes to the distribution as activity evolves over time. D-F: Activity from the first day after synaptic potentiation in Fig 2E was divided between epochs of high frequency activity and the epochs of lower activity. The numbers above Fig 2E define the epochs of activity presented here. A new peak in the distribution of the emerges around 100ms. This peak corresponds to firing rates in the range of rat hippocampal theta activity and is highly associated with hippocampal learning and memory (35). G-H: Activity from the fourth day after treatment in Fig 2E was divided between epochs of high frequency activity and the epochs of lower activity. This 100 ms peak in the distribution is still present. Note the y-axis scale change on day 5 (panels G and H).

**Fig 12:**
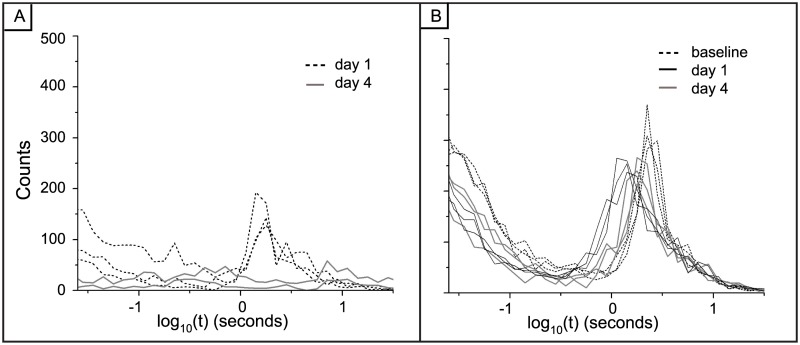
Interspike (ISI) distributions for low activity epochs during synaptic potentiation and vehicle are similar to the baseline, pre-treatment distributions. A) Log histograms for epochs during which the activity is low after synaptic potentiation. For day 1 these correspond to epochs 2, 4 and 6 from Fig 2E and on day 4 these are epochs 1 and 3 from Fig 2F. B) Log histograms of inter-spike intervals from vehicle networks.

As stated above, the synaptic potentiation treatment caused the spiking activity to cluster into periods of high activity as seen in the raster plots of Figs 2 and 3. Therefore, to investigate possible changes in inter-spike interval distributions, we created epochs that corresponded to these distinct periods of activity and the epochs are numbered in Fig 2E and 2F. We generated log histograms (Figs 11 and 12A) of the inter-spike intervals (ISI) from each epoch in these raster plots and compared them to log histograms of ISIs from the raster plots of the vehicle networks in Figs 2B, 2C and 12B). Since the activity in the vehicle networks was not differentiated over time, the periods for the vehicle epochs were equally divided.

Fig 11 displays the histograms of ISIs for the pre-treatment synaptic potentiation activity along with the ISIs for the high frequency activity on day 1 (epochs 1,3 and 5) and day 4 (epochs 2 and 4). After treatment, there is a reorganization of the distribution of inter-spike intervals as it shifts from bi-modal to log-normal with most of the activity taking place within short intervals. Additionally, a potentially novel dynamical marker of LTP appears; there is an induced peak in the distribution at approximately 100ms, independent of the network-wide firing rate (black colored bin in Fig 11D–11H). This corresponds to an interval in the theta range, i.e., 4–9 Hz band, commonly seen after LTP induction within *in vivo* experiments [36]. This theta peak is not present within the ISI distributions from the vehicle networks (Fig 12B). Lastly, the reorganization of inter-spike intervals to a log-normal distribution does not occur in the vehicle networks (Fig 12B). The bi-modal nature of the vehicle distributions is time-independent as it is present during all periods of recording.

**Fig 13:**
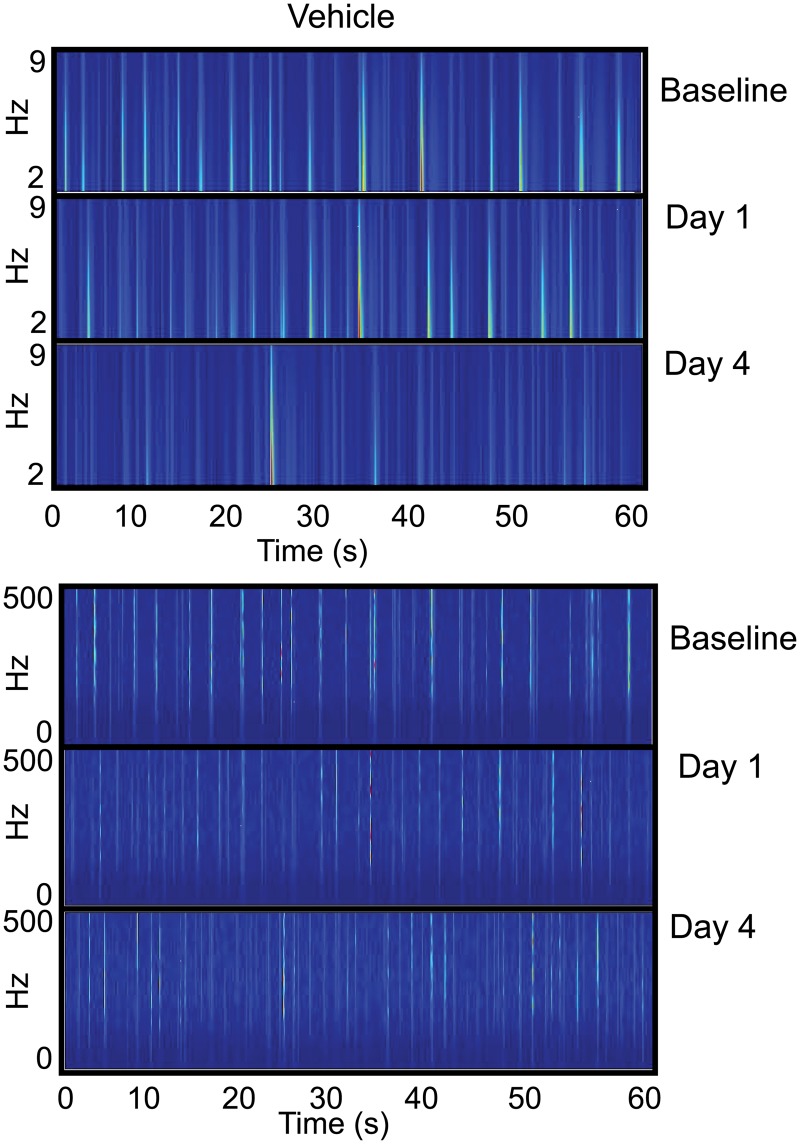
Vehicle networks do not show large power distributions within low- and high-frequency networks. Short time, Fourier transform based time-frequency analyses were performed on one-minute epochs of low-pass filtered (top set) and high-pass filtered (bottom set) data from one representative electrode. Each set is a montage of three images in which the top image corresponds to baseline activity, the middle image corresponds to activity one day after treatment and the bottom image is the activity that was recorded four days after treatment. Within each image are vertical striations in which each striation is a color map that represents the power of the frequency present within the electrode. Red corresponds to high power and blue is low power. In both the low-pass filtered and high-pass filtered montages, the activity does not appreciably change before and after treatment and the power within each frequency regime is low.

**Fig 14:**
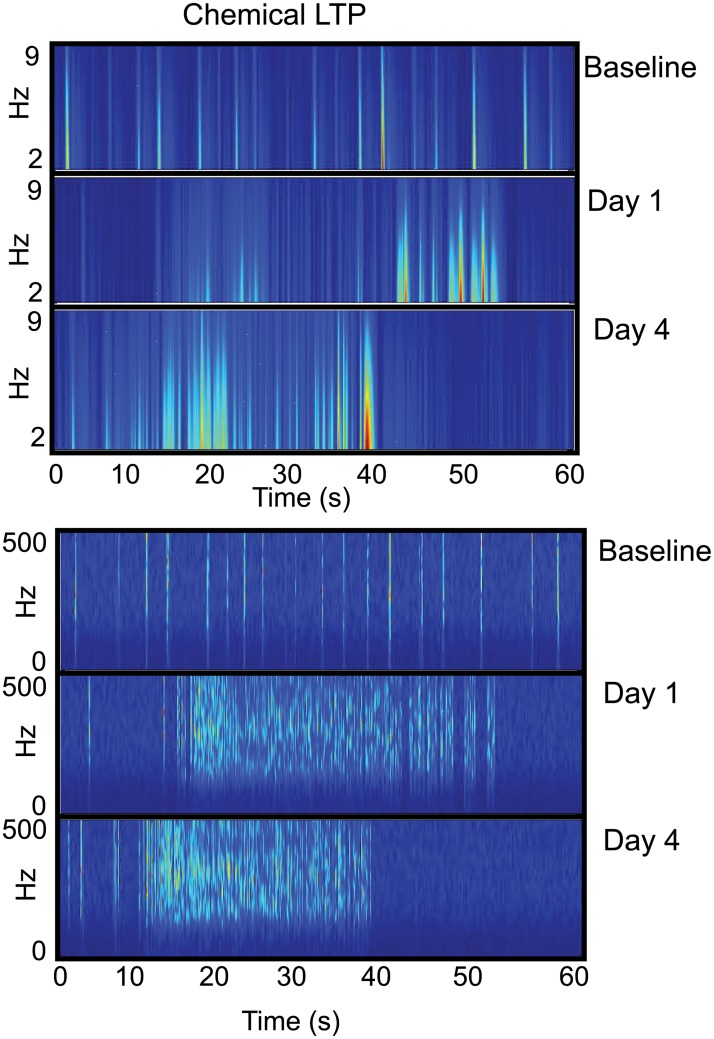
Synaptically potentiated networks display changes in power distributions after treatment. Short time, Fourier transform based time-frequency analyses were performed on one-minute epochs of low-pass filtered (top set) and high-pass filtered (bottom set) data from one representative electrode. Each set is a montage of three images in which the top image corresponds to baseline activity, the middle image corresponds to activity one day after treatment and the bottom image is the activity that was recorded four days after treatment. Within each image are vertical striations in which each striation is a color map that represents the power of the frequency present within the electrode. Red corresponds to high power and blue is low power. There is a spread in the sub-threshold oscillations (top set) one day after treatment and persists four days later. These low-theta oscillations also increase in their power four days after treatment. Within the high-frequency regime (bottom set), there is persistent activity one day and four days after treatment. The power distribution within the vertical striations is similar to that seen in the baseline activity, with the large increase in frequency that confirms Figs 2–4.

These histograms remove the dependence of frequency with time since they solely display the number of ISIs collected during the recording epochs. This allows us to assess the stability of the distribution of ISIs before and after synaptic potentiation as well as within the vehicle-treated networks. However, this does not provide us with a time course of oscillatory activity with its associated power spectra. Therefore, to investigate the frequency components observed over time, we performed a short time, Fourier transform (STFT) based time-frequency analysis on the collected data. Whereas the raster plots of Figs 2–4 display the high-frequency spiking activity, Figs 13 and 14 presents waveforms for both low-pass and high-pass filtered data from representative electrodes during a one-minute segment of this activity. This allows us to study the emergence of collective phenomena rather than focusing on the dynamical features of single spikes and bursts that occur on a shorter time scale. The low-pass filtered data represents the subthreshold activity and the high-pass filtered data is a result of the spiking activity within the network. Each figure is composed of two sets of images with the top set corresponding to the low-pass filtered data and the bottom set corresponding to the high-pass filtered data. Each set is a montage of three images in which the top image corresponds to baseline activity, the middle image corresponds to activity one day after vehicle or treatment and the bottom image is the recorded activity on day 4. Lastly, each image is a color-coded time series of activity in which each vertical striation is a color map representing the power (red—high power and blue—low power) of the given frequency (as defined by the y-axis) that is present as a function of time. Data from the vehicle networks are shown in Fig 13 with the corresponding data from the synaptic potentiation treatment presented in Fig 14.

In the low-pass filtered, vehicle-treated networks of Fig 13, there is little change in activity between the baseline and day 1. The power is also low within the frequency range as depicted by the largely pale blue streaks indicating a small presence of subthreshold activity. On day 4, the power distribution within the frequency range is lower as the pale blue streaks continue to fade. This pattern of low power is also present in the high frequency domain, again with little change from baseline to days 1 and 4 indicating that the spikes are distinct in time, punctuated by periods of quiescence. In the treated networks of Fig 14, both baseline patterns in the low- and high-pass filtered data display similar trends seen in the vehicle networks. However, there are marked differences on days 1 and 4 within both regimes. In the low-pass filtered montage, there is an increase in the power at the low frequencies and these periods of low frequency activity are longer in duration—as indicated by the spread—than in the vehicle networks. This pattern continues on day 4 with sustained low frequency activity over several seconds in duration. Riding on top of these sub-threshold oscillations is a barrage of spiking activity, as shown in the high-pass filtered montage. The frequency is very high at the onset of the barrage with some frequency attenuation as time progresses. However, the frequency of spiking does not decrease to that seen in the vehicle networks. Lastly, we note that the increase of the low frequency, i.e., the spread in activity occurs both at the beginning and at the tail end of the spiking activity. This appears one day after treatment and becomes more pronounced four days later.

## Discussion

We report long time-scale dynamical results from an *in vitro* network of hippocampal neurons after a synaptic potentiation treatment. We recorded electrical activity from these networks up to five days after treatment. There was a large increase in the Fano factor after synaptic potentiation that reflects the increase in variability in activity; the networks alternated between states of high, bursting epochs and periods of lower activity. The control networks did not show a change in the Fano factor during development suggesting that network maturation alone does not significantly impact the variability of overall spiking activity. Additionally, despite the fact that mechanical perturbations may have caused a decrease in network activity in the vehicle cultures, this perturbation did not affect spike regularity as the Fano factor was distributed along the identity line as in the control networks.

We show that while the effects of potentiation continued to modulate network dynamics after the removal of pharmacological agents, regulatory mechanisms seems to prevent the network from transitioning into a state of unbridled excitation with a rampant increase in the number of overall spikes. However, it is clear that there are a host of possible biochemical mechanisms that may be involved. We suggest that the initial potentiation may induce depression on a subset of synapses, resulting in the decrease of activity that we observe. However, the focus of this study was to investigate the network effects that culminate from an initial synaptic perturbation and future work will entail elucidating the underlying biochemical mechanisms. We show that increase in global spiking activity was maintained up to one day after treatment but returned to normal levels within four days thereafter. In addition, there is a uniform, symmetric expansion in the spike count distributions one and four days after treatment. This expansion takes the shape of an ellipsoid with the principle axis of rotational symmetry following the identity line. This suggests that a conservation mechanism may be present that will maintain a balanced state of activity after the chemical perturbation, i.e., any increase in activity in a set of electrodes is offset by a decrease in activity in others, as we have observed.

Next, the synaptic potentiation networks display a strong, persistent theta peak in the log histograms of inter-spike intervals (ISIs). This is intriguing because theta range activity is detected in the hippocampus during learning tasks in animals [36–39] as well as in the prefrontal cortex of humans [40, 41, 42]. We previously reported on this 100 ms peak after synaptic potentiation 20 minutes after induction and these current studies indicate that this peak persists over a longer period of time. Notably however, the ISI distributions acquired in the short-term studies had two peaks in the short ISI regime whereas in these long-term recordings the 100ms bin is the sole peak within the distribution. We also note that the distribution of ISIs reported in the epochs between the super-bursts as well as within the vehicle networks are characteristic of our previously published studies.

We speculate that this theta interval may be a resonant interval over which a subset of the network is responsive. It has been previously proposed that bursts having certain resonant inter-spike frequencies increase the likelihood of a postsynaptic response over bursts with higher or lower frequencies [43]. A resonance effect could therefore explain why some electrodes increase their activity while activity in other electrodes decrease, allowing the network to operate under a spike conservation principle.

We also note the increased prominence of subthreshold, low frequency oscillations that manifest one day and persist for four days after synaptic potentiation. These oscillations are within the theta regime and their largest spectral power appears at the end of a spiking barrage. While our studies were not performed on an intact structure such as a slice or *in vivo*, we suggest that the generation of these theta oscillations, after a synaptic potentiation protocol, within a network without native architecture might indicate the presence of a fundamental dynamical pattern of hippocampal networks.

Lastly, while these studies were performed on a cultured preparation, we must be very prudent in making direct connections to *in vivo* systems. Nevertheless, our goal was to study the long-term effects of potentiating a large fraction of synapses within a network—since many *in vivo* synapses are indeed potentiated during a learning exercise. To this end, we asked the question: “Does LTP beget more LTP?”, because the understanding of LTP is that it increases the likelihood that the postsynaptic neuron will fire an action potential. This, therefore, led us to wonder whether within a network of neurons, would chemical LTP drive the network towards an unstable, hyper-excited state? As this is not what we observed over time, we suggest that the healthy neural circuit does not evolve into a pathological state when the excitatory-inhibitory balance is moderately perturbed; there may be homeostatic mechanisms that prevent this behavior from taking place. However, in diseased circuits in which the excitatory-inhibitory balance has been perturbed, it may be that the underlying mechanisms normally present to “rein in” the network may have been damaged.

## Conclusions

We demonstrate that synaptic potentiation causes a persistent elevation of network activity but not in a pathological manner. Hyper-excitability may be constrained by a conservation of activity principle that is governed by the steady-state regulation of the network.
